# Altered functional activity and connectivity in Parkinson’s disease with chronic pain: a resting-state fMRI study

**DOI:** 10.3389/fnagi.2025.1499262

**Published:** 2025-03-03

**Authors:** Erlei Wang, Nan Zou, Jinru Zhang, Yiqing Bao, Yang Ya, Junkang Shen, Yujing Jia, Chengjie Mao, Guohua Fan

**Affiliations:** ^1^Department of Radiology, The Second Affiliated Hospital of Soochow University, Suzhou, China; ^2^Department of Radiology, Nanjing TCM Hospital Affiliated to Nanjing University of Traditional Chinese Medicine, Nanjing, China; ^3^Department of Neurology and Clinical Research Center of Neurological Disease, The Second Affiliated Hospital of Soochow University, Suzhou, China

**Keywords:** Parkinson’s disease, chronic pain, resting-state fMRI, the amplitude of low-frequency fluctuations, functional connectivity

## Abstract

**Background:**

Chronic pain is a common non-motor symptom of Parkinson’s disease (PD) that significantly impacts patients’ quality of life, but its neural mechanisms remain poorly understood. This study investigated changes in spontaneous neuronal activity and functional connectivity (FC) associated with chronic pain in PD patients.

**Methods:**

The study included 41 PD patients with chronic pain (PDP), 41 PD patients without pain (nPDP), and 29 healthy controls. Pain severity was assessed using the visual analog scale (VAS). Resting-state fMRI images were used to measure the amplitude of low-frequency fluctuations (ALFF) as an indicator of regional brain activity. Subsequently, FC analysis was performed to evaluate synchronization between ALFF-identified regions and the entire brain.

**Results:**

Compared to nPDP patients, PDP patients exhibited decreased ALFF in the right putamen, and increased ALFF in motor regions, including the right superior frontal gyrus/supplementary motor area and the left paracentral lobule/primary motor cortex. Additionally, PDP patients exhibited diminished right putamen-based FC in the midbrain, anterior cingulate cortex, orbitofrontal cortex, middle frontal gyrus, middle temporal gyrus, and posterior cerebellar lobe. The correlation analysis revealed that ALFF values in the right putamen were negatively associated with VAS scores in PDP patients.

**Conclusion:**

This study demonstrates that chronic pain in PD is associated with reduced ALFF in the putamen and disrupted FC with brain regions involved in pain perception and modulation, highlighting the critical role of dopaminergic degeneration in the development and maintenance of pain in PD.

## Introduction

Parkinson’s disease (PD) is characterized by the progressive degeneration of the nigrostriatal dopamine pathway, primarily leading to motor impairments (Eggers et al., 2020). However, a range of non-motor symptoms is also commonly observed in PD (Chaudhuri and Schapira, 2009). Chronic pain in PD is a disabling and heterogeneous symptom that significantly impairs patients’ quality of life. It has been reported that approximately 62 to 91% of PD patients experience chronic pain (Negre-Pages et al., 2008; Buhmann et al., 2017), which is more common in women and in the later stages of the disease, often accompanied by other non-motor symptoms, such as sleep disturbances (Ghosh et al., 2020). Despite its clinical significance, chronic pain in PD remains inadequately managed due to a limited understanding of its underlying mechanisms.

Multiple lines of evidence suggest that dopaminergic degeneration may play a crucial role in the development and maintenance of pain in PD. First, in pain-free PD patients exposed to pain stimuli, functional studies revealed abnormal brain activation in regions such as the somatosensory cortex, insula, prefrontal cortex, anterior cingulate cortex, cerebellum, and lower pons, with partial reversal following levodopa administration (Brefel-Courbon et al., 2013; Tan et al., 2015; Tessitore et al., 2017). These findings suggest central sensitization in PD, which may be driven by dopaminergic dysfunction. Second, aberrant resting-state functional connectivity (FC) of the nucleus accumbens, a key component of the mesolimbic dopaminergic pathway, has been linked to the onset of pain during on-to-off periods in PD (Kinugawa et al., 2022). Third, positron emission tomography studies have linked dopaminergic denervation in subcortical areas, such as the caudate nucleus, and cortical regions, including the insula and posterior cingulate cortex, to musculoskeletal pain and altered subjective heat pain thresholds in PD patients (Dellapina et al., 2019; Rukavina et al., 2023). Taken together, these studies suggest that pain in PD is associated with abnormal functional changes in brain regions involved in pain processing and modulation, potentially influenced by dopamine deficits.

The amplitude of low-frequency fluctuations (ALFF) is a resting-state fMRI metric that measures the amplitude of spontaneous brain oscillations, reflecting regional neural activity (Yu-Feng et al., 2007). FC refers to the synchronized neural activity patterns across distinct brain regions (Biswal et al., 1995). Currently, only a limited number of studies have explored the changes in ALFF and FC associated with chronic pain in PD. For example, abnormal fractional ALFF has been observed in the frontal inferior orbital region, temporal inferior areas, and cerebellum, as well as disrupted connectivity between the nucleus accumbens and hippocampus in PD patients with persistent pain (Polli et al., 2016). Additionally, a recent study revealed an abnormal connectivity pattern between the raphe nuclei and pain-related brain regions in PD patients with chronic pain (Shen et al., 2022). However, previous studies have independently examined ALFF and FC changes related to chronic pain in PD patients, often with small sample sizes. The combined analysis of ALFF and FC enables researchers to assess both the activation of individual brain regions and their inter-regional synchronization, providing a more comprehensive understanding of the neural mechanisms of chronic pain in PD.

In this study, we aimed to explore whole-brain alterations in ALFF and corresponding FC associated with chronic pain in 41 PD patients (PDP), 41 PD patients without pain (nPDP), and 29 normal controls (NCs) using resting-state fMRI data. First, we compared ALFF values among the three groups to assess spontaneous brain activity. Second, whole-brain FC for seed regions identified through ALFF analysis was calculated and compared across the three groups. Third, we examined the correlations between the alterations in ALFF and FC and the Visual Analog Scale (VAS) pain scores in PDP patients. As previous studies have highlighted the association between dopaminergic degeneration in cortical and subcortical regions and pain in PD, as well as their critical roles in different dimensions of pain processing—such as the sensory-discriminative (e.g., putamen and posterior insula), emotional/affective (e.g., ventral tegmental area, nucleus accumbens, anterior cingulate cortex, and anterior insula), and cognitive modulation (e.g., prefrontal cortex)—we hypothesized that PDP patients would exhibit altered ALFF and FC in these dopaminergic regions, including the midbrain, striatum, insula, anterior cingulate cortex, and prefrontal cortex (Scott et al., 2006; De Ridder et al., 2021).

## Methods

### Participants

A total of 131 participants, comprising 97 PD patients and 34 NCs, were recruited from the Second Affiliated Hospital of Soochow University between September 2020 and March 2022. PD diagnoses were made by an experienced movement disorder neurologist based on the British Parkinson’s Disease Society Brain Bank diagnostic criteria (Hughes et al., 1992). Exclusion criteria were: (1) pain from other causes (e.g., fractures, herpes zoster, cancer); (2) a history of traumatic brain injury or neurosurgery; (3) severe psychiatric or neurological comorbidities; and (4) contraindications to MRI. NCs were included if they had no history of neuropsychiatric disorders or pain symptoms.

The Unified Parkinson’s Disease Rating Scale motor section (UPDRS-III) (Fahn and Elton, 1987) and the Hoehn and Yahr staging scale (H-Y) (Hoehn and Yahr, 1967) were used to evaluate motor impairments and disease severity. Pain intensity was measured using the Visual Analogue Scale (VAS), which ranges from 0 (no pain) to 10 (severe pain) (Price et al., 1983). Cognitive function was assessed using the Mini-Mental State Examination (MMSE) and the Montreal Cognitive Assessment (MoCA) (Nasreddine et al., 2005). Depression and anxiety levels were evaluated with the Hamilton Depression Rating Scale (HAMD) (Hamilton, 1967) and the Hamilton Anxiety Rating Scale (HAMA) (Hamilton, 1959), respectively. The study was approved by the Medical Ethical Committee of the Second Affiliated Hospital of Soochow University, and Written informed consent was obtained from all participants.

### MRI data acquisition

MRI data were acquired using a 3.0 T MRI scanner (Siemens, Prisma, Germany) equipped with a 64-channel head and neck coil. Participants were instructed to close their eyes, remain awake, and hold their head still. Tight foam padding was used to minimize head movement, and earplugs were used to reduce noise. Resting-state fMRI images were acquired using an echo planar imaging pulse sequence with the following imaging parameters: repetition time (TR) = 1,240 ms, echo time (TE) = 32 ms, field of view (FOV) = 215 mm × 215 mm, slice thickness = 2.5 mm, flip angle (FA) = 67°, matrix size = 86 × 86, slices = 57, and 300 time points. A sagittal Three-dimensional magnetization-prepared rapid-acquisition gradient echo sequence structural sequence was acquired with the following parameters: TR = 2,300 ms, TE = 2.34 ms, FA = 8°, FOV = 256 mm × 256 mm, slice thickness = 1.0 mm, number of slices = 240, matrix size = 256 × 256, voxel size = 1 mm × 1 mm × 1 mm. To minimize the effect of medications on the experimental results, all PD patients discontinued their PD medications 12 h prior to the scan.

### Data preprocessing

The resting-state fMRI data were analyzed by the Data Processing & Analysis of Brain Imaging (DPABI_V6.0)^1^ toolbox implemented on the MATLAB 2018b platform. The steps were summarized as follows: (1) conversion of data from DICOM files to NIFTI format; (2) removal of the first 10 time points to avoid magnetic saturation effects; (3) slice timing correction; (4) head motion correction (exclusion criteria: displacement >2 mm or angular rotation >2); (5) co-registration, spatial normalization, and resampling to 3 × 3 × 3 mm^3^ resolution; (6) spatial smoothing using a 6 mm full-width at half-maximum (FWHM) Gaussian kernel; (7) removal of covariates (regression of signals from 24 head movement parameters, cerebral white matter, and cerebrospinal fluid).

### ALFF analysis

Individual voxel-wise ALFF maps were generated using DPABI software. A fast Fourier transform was applied to convert the whole-brain voxel-wise time series from the time domain to the frequency domain, obtaining the power spectrum. Temporal band-pass filtering (0.01–0.08 Hz) was then applied to produce ALFF maps, which were subsequently standardized into zALFF maps using z-score transformation for further analysis.

### Seed-based FC analysis

Based on the ALFF results, we performed seed-based FC analysis. The DPABI software was used to generate individual seed-to-voxel FC maps through several steps. First, brain regions showing significant ALFF differences between the PDP and nPDP groups [the right putamen, right superior frontal gyrus (SFG)/supplementary motor area (SMA), and left paracentral lobule (PCL)/primary motor cortex (M1) were saved as seed masks]. Then, the average time series of all voxels within each seed region were extracted and correlated with the time series of every voxel across the whole brain using Pearson correlation. Finally, Fisher’s r-to-z transformation was performed to normalize the correlation coefficients, resulting in the FC maps.

### Statistical analysis

Demographic and clinical data were analysed using SPSS 20.0 software. The Shapiro Wilk test was used to evaluate the normality of the data. For variables with a normal distribution, one-way ANOVA and two-samples *t*-tests were conducted. Non-normally distributed variables were analyzed using the Kruskal–Wallis H test and the Mann–Whitney U test. The chi-square test was performed to compare categorical variables, including sex. The significance level was set at *p* < 0.05.

Statistical analyses of the imaging data were conducted following the guidelines of the DPABI statistical module. A one-way ANOVA was performed to assess group differences in ALFF and FC maps among the three groups, with sex, age, years of education, and head movement (mean framewise displacement) as covariates. Specifically, DPABI provides *post-hoc* comparison corrections for group pairs following ANOVA. Bonferroni correction was applied to these post hoc comparisons. The resulting p maps were converted to z maps, reflecting the direction of group mean differences. These z maps were corrected for multiple comparisons using Gaussian Random Field correction (GRF), with voxel-level *p* < 0.005 and cluster-level *p* < 0.05, two-tailed (Cao et al., 2022; Chang et al., 2022; Zhao et al., 2022).

Additionally, the average ALFF and FC values of brain regions with significant differences between the PDP and nPDP groups were extracted using the DPABI software. Partial correlation analysis was then performed with SPSS 20.0 to assess the relationship between these values and VAS scores in the PDP group, controlling for age, sex, disease duration, UPDRS-III, HAMA, and HAMD. A significance threshold of *p* < 0.05 was used.

## Results

### Demographic and clinical data of participants

A total of 20 participants, including 15 PD patients and 5 NCs, were excluded due to excessive head motion, intracranial lesions, or other imaging artifacts. Consequently, 82 PD patients and 29 NCs were included in the final analysis. Of these, 41 PD patients with VAS scores >0 and pain lasting >3 months were assigned to the PDP group, while the remaining 41 with scores of 0 were assigned to the nPDP group. The detailed demographic and clinical data of the PDP, nPDP, and NCs groups are presented in Table 1. No significant differences were observed in age, sex, education level, or MMSE scores across the three groups. Furthermore, the duration of disease, H-Y stage, UPDRS-III, LEDD, MoCA, HAMA, and HAMD scores did not significantly differ between the PDP and nPDP groups.

**Table 1:** Demographic and clinical data of the three groups.

	PDP (*n* = 41)	nPDP (*n* = 41)	NCs (*n* = 29)	Statistical values	*p-*value
Age (year)	63.2 ± 9.6	63.1 ± 11.6	60.2 ± 5.8	0.977	0.380^a^
Sex (M/F)	22/19	24/17	15/14	0.363	0.834^b^
Education (year)	9.22 ± 4.7	7.7 ± 4.2	6.9 ± 4.7	2.555	0.082^a^
Duration (month)	42.5 (14.3, 60)	22 (12.5, 36)		1.131	0.258^c^
UPDRS III	20 (13, 28)	17 (9, 22)		1.373	0.170^e^
H-Y	2 (1.5, 2.5)	1.5 (1, 2.5)		0.918	0.359^e^
LEDD	300 (75, 500)	300 (0, 500)		0.879	0.379^e^
MMSE	28(24.5, 29)	26 (23.25, 27.75)	26 (25, 28.5)	2	0.052^d^
MoCA	22.5 ± 4.5	20.47 ± 4.4		1.463	0.151^c^
HAMD	7.5 (1, 12.8)	4 (1, 9)		−1.845	0.650^e^
HAMA	7.5 (5, 11.2)	5 (3, 9.8)		−1.202	0.229^e^
VAS	6 (4, 7)	0		0.936	0.001^e^

Values are shown as mean ± SD, or median (interquartile range); PDP, PD patients with chronic pain; nPDP, PD without pain; NCs, normal controls; n, number; M, male; F, female; UPDRS, Unified Parkinson’s Disease Rating Scale; H-Y, Hoehn and Yahr stage; LEDD, levodopa equivalent daily dose; MMSE, Mini-Mental State Examination; MOCA, Montreal Cognitive Assessment; HAMD, Hamilton Depression Scale; HAMA, Hamilton Anxiety Rating Scale; VAS, Visual Analog Scale. ^a^One-way analysis of variance. ^b^Chi-square test. ^c^Two-sample *t*-test. ^d^Kruskal-Wallis H test. ^e^Mann-Whitney U test.

### ALFF results

**Figure 1:**
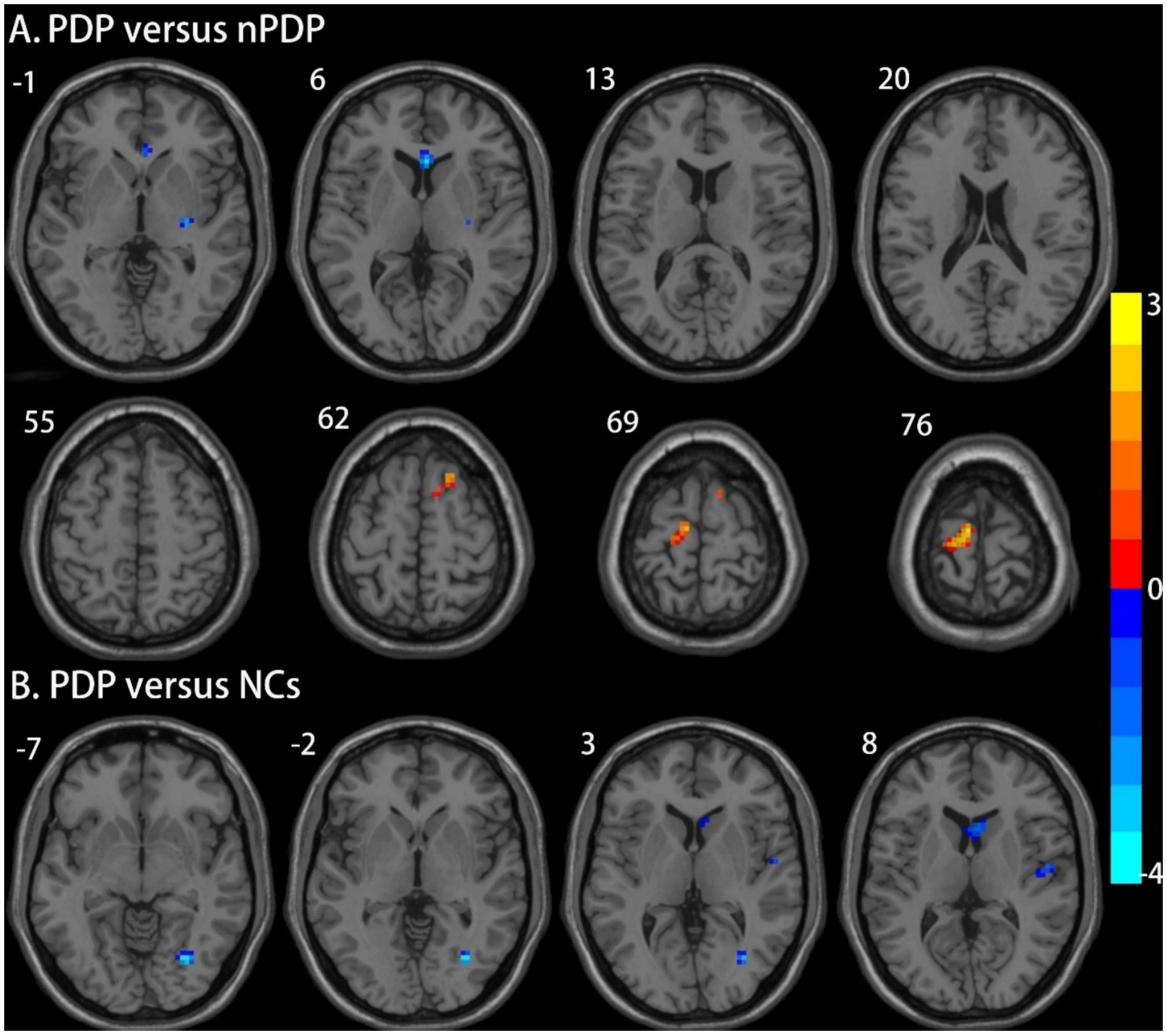
Brain regions showing significant differences in ALFF between the three groups. **(A)** Compared to the nPDP group, the PDP group exhibited decreased ALFF in the right putamen and increased ALFF in the right superior frontal gyrus/supplementary motor area and the left paracentral lobule/primary motor cortex; **(B)** The PDP group also exhibited decreased ALFF in the right fusiform and right primary motor cortex compared to NCs. The results were reported at a voxel-level *p* < 0.005 and a cluster-level *p* < 0.05, as determined by GRF correction. The color bar represents Z-values.

Compared to the nPDP group, the PDP group exhibited decreased ALFF in the right putamen and increased ALFF in the right SFG/SMA and the left PCL/M1 (Table 2 and Figure 1A). The PDP group also exhibited decreased ALFF in the right fusiform and right M1 compared to NCs (Table 2 and Figure 1B). No brain regions with significant differences in ALFF were observed in the nPDP group relative to NCs.

**Table 2:** ALFF differences between PDP, nPDP and NCs groups.

Brain region	L/R	Coordinates MNI			Cluster size	*Z* value
		x	y	z		
PDP vs. nPDP						
Putamen	R	30	−15	15	15	−3.855
Corpus Callosum		3	24	6	32	−3.747
SFG/SMA	R	18	18	60	27	3.675
PCL/M1	L	−9	−12	72	62	3.969
PDP vs. NCs						
Fusiform	R	30	−69	−6	37	−4.279
M1	R	51	−9	12	22	−3.447

ALFF, amplitude of low-frequency fluctuation; PDP, PD patients with chronic pain; nPDP, PD without pain; NCs, normal controls; MNI, Montreal Neurological Institute; SFG, superior frontal gyrus; SMA, supplementary motor area; PCL, paracentral lobule; M1, primary motor cortex.

### FC results

**Figure 2:**
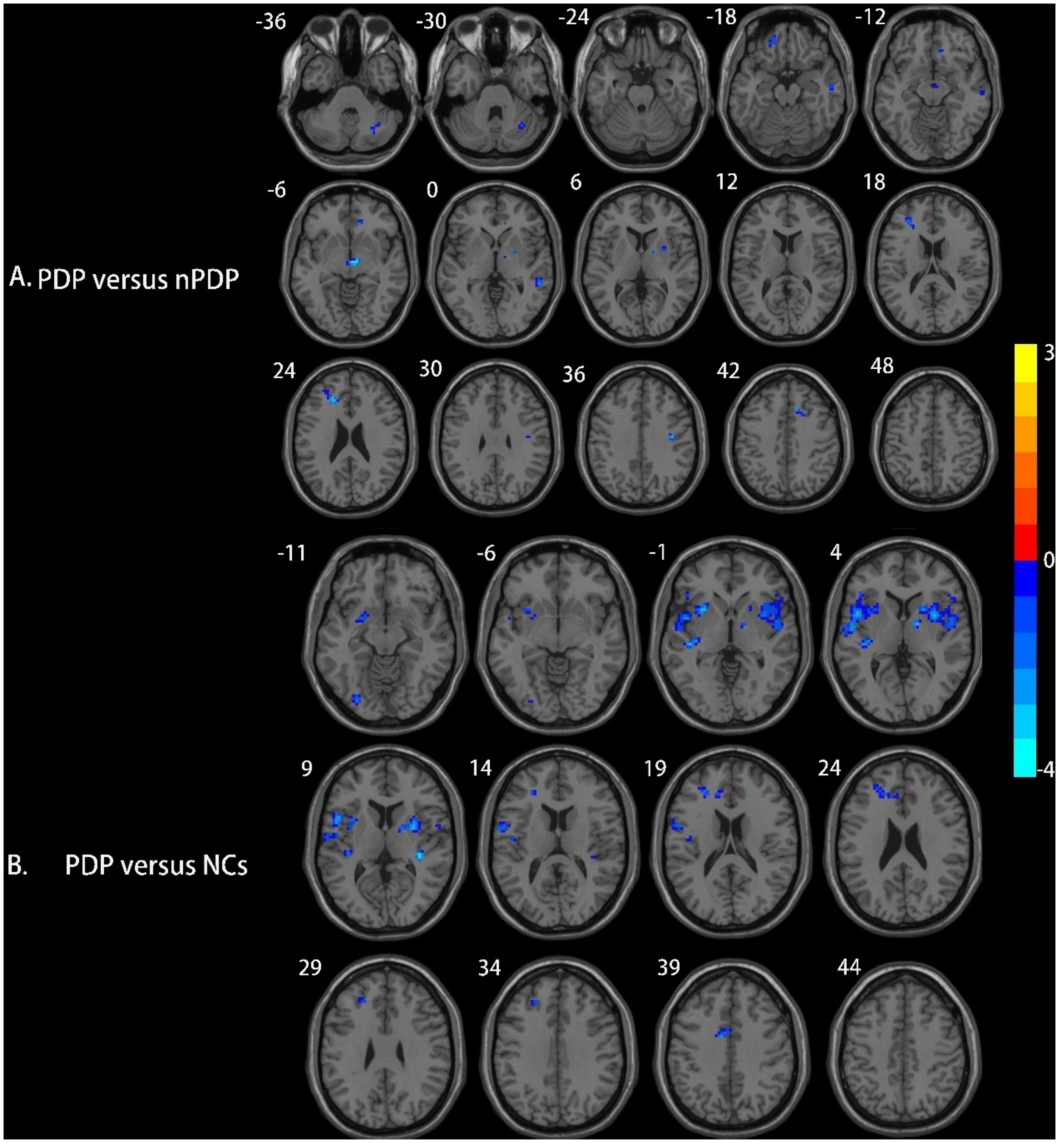
Brain regions showing differences in right putamen-based FC among the three groups. **(A)** Compared to the nPDP group, the PDP group exhibited significantly decreased FC between the right putamen and the midbrain, right anterior cingulate cortex, left orbitofrontal cortex, left middle frontal gyrus, right middle temporal gyrus, and right posterior cerebellar lobe; **(B)** compared to the NCs, the PDP group exhibited decreased FC of the right putamen with the left insula, right insula, left middle frontal gyrus, and left superior temporal gyrus. Blue represents brain regions with decreased FC (voxel-level *p* < 0.005, cluster-level *p* < 0.05, as determined by GRF correction). The color bar represents Z-values.

Compared to the nPDP group, the PDP group showed decreased right putamen-based FC in the midbrain, right anterior cingulate cortex (ACC), left orbitofrontal cortex (OFC), left middle frontal gyrus (MFG), right middle temporal gyrus (MTG), and right posterior cerebellar lobe (Table 3 and Figure 2A). No significant FC changes based on the right SFG/SMA or left PCL/M1 were observed in the PDP group compared to the nPDP group. Compared to the NCs, the PDP group exhibited decreased right putamen-based FC in the left insula, right insula, left MFG, and left superior temporal gyrus (STG) (Table 3 and Figure 2B).

**Table 3:** FC differences between PDP, nPDP and NCs group.

ROI	Brain region	L/R	Coordinates MNI			Cluster size	*Z* value
			x	y	z		
Putamen_ R							
PDP vs. nPDP	Midbrain		9	−12	−6	55	−4.055
	ACC	R	12	36	−3	15	−3.588
	OFC	L	−15	42	−21	19	−3.335
	MFG	L	−21	33	24	62	−3.888
	Cerebellum	R	27	−66	−36	26	−3.334
	MTG	R	54	−39	0	19	−3.473
PDP vs. NCs	Insula	L	−30	12	−3	312	−4.295
	Insula	R	30	9	6	273	−4.042
	MFG	L	−27	39	18	76	−3.477
	STG	L	−42	−24	0	43	−3.878

PDP, PD patients with chronic pain; nPDP, PD without pain; NCs, normal controls; MFG, middle frontal gyrus; OFC, Orbito-frontal cortex; MTG, middle temporal gyrus; ACC, anterior cingulate cortex; STG, superior temporal gyrus.

### Correlation analysis results

**Figure 3:**
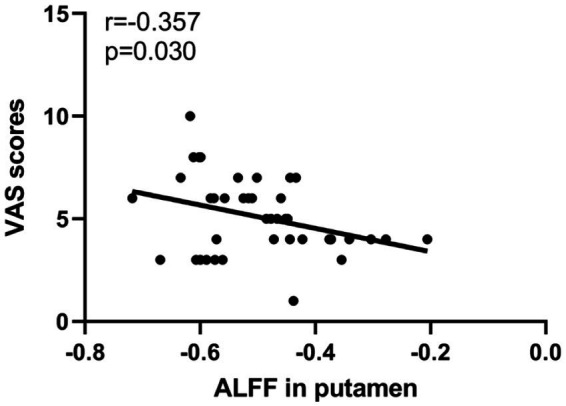
Scatter plots of partial correlation between ALFF values in the putamen and VAS scores in PDP patients.

The correlation analysis revealed a negative correlation between ALFF values in the right putamen and VAS scores in PDP group (*r* = −0.357, *p* = 0.030; Figure 3).

## Discussion

This study investigated changes in ALFF and FC associated with chronic pain in PD patients using resting-state fMRI data. The key findings are as follows: First, compared to nPDP patients, PDP patients exhibited decreased ALFF in the right putamen and increased ALFF in the right SFG/SMA and left PCL/M1. Second, PDP patients showed significantly decreased right putamen-based FC in multiple dopaminergic regions, including the midbrain, right ACC, left OFC, left MFG, right MTG, and right posterior cerebellar lobe. Third, a significant negative correlation was observed between ALFF values in the right putamen and VAS scores in PDP patients. These findings suggest that chronic pain in PD is associated with abnormal ALFF and FC in dopaminergic regions involved in pain perception and modulation, possibly driven by degeneration within the nigrostriatal-cortical dopaminergic pathway.

PDP patients exhibited reduced ALFF in the right putamen compared to nPDP patients, suggesting that decreased neuronal activity in this region may contribute to the development and maintenance of pain in PD. This finding aligns with previous studies identifying structural and functional changes in the putamen in various chronic pain conditions, such as complex regional pain syndrome, ankylosing spondylitis, and migraine (Gao et al., 2016; Azqueta-Gavaldon et al., 2020; Hua et al., 2020). Notably, we observed a significant negative correlation between mean ALFF values in the right putamen and VAS scores in PDP patients, indicating a potential role of the putamen in encoding pain intensity. This result is consistent with prior research demonstrating that dopamine D2 receptor binding potential in this region correlates with pain intensity ratings induced by nociceptive stimuli in healthy individuals (Hagelberg et al., 2004). Beyond its motor functions, the putamen is directly involved in the sensory aspects of pain (Scott et al., 2006; Starr et al., 2011). As a critical relay station between the cortex and substantia nigra, the putamen is essential for pain modulation, a process primarily mediated by dopamine D2 receptors (Hagelberg et al., 2004). Therefore, our findings suggest that chronic pain in PD may be associated with dopaminergic degeneration in the nigrostriatal-cortical pathway.

PDP patients exhibited increased ALFF in motor regions, including the M1, PCL, SMA, and SFG. These findings align with previous studies showing increased excitability and gray matter volume in M1 of chronic pain populations, such as trigeminal neuralgia, fibromyalgia, and chronic low back pain (Binshtok et al., 2013; Castillo Saavedra et al., 2014; Zhang et al., 2019). Furthermore, recent studies using non-invasive brain stimulation have shown significant analgesic effects from motor cortex excitation (Maarrawi et al., 2007; de Andrade et al., 2011; Kandić et al., 2021). Although M1 is not a component of the pain matrix, it is thought to play a crucial role in pain modulation through its feedback loop with pain-related regions (Castillo Saavedra et al., 2014). Therefore, considering the reduced ALFF in the putamen and its strong connectivity with motor regions, the increased ALFF in motor regions may indicate ineffective compensatory mechanisms attempting to suppress pain in PDP patients.

Compared to the nPDP group, the PDP group showed decreased right putamen-based FC in the midbrain, right ACC, left OFC, left MFG, right MTG, and posterior cerebellum. This indicates that reduced activity in the putamen may affect the activity of multiple brain regions involved in pain perception and modulation. This finding is consistent with previous research showing that patients with putamen lesions exhibit decreased pain-induced activation in these nociceptive processing regions (Starr et al., 2011). Furthermore, probabilistic tractography analyses in healthy individuals provide additional evidence linking the pain-activated putamen to pain-related regions such as the ACC, insula, middle frontal gyrus, and midbrain in this study (Starr et al., 2011).

Specifically, The VTA and SN in the midbrain, as primary sources of dopamine, play a pivotal role in pain perception and modulation through extensive dopaminergic projections to subcortical and cortical regions, including the putamen, ACC, MFC, OFC, and cerebellum (Borsook et al., 2010; Benarroch, 2022). The ACC, a key structure in the medial pain system, is predominantly involved in processing the affective component of pain (De Ridder et al., 2021). Previous studies have reported abnormal pain-induced activation in the ACC in PDP patients compared to nPDP patients, consistent with our findings (Brefel-Courbon et al., 2013). The MFC and OFC, as components of the PFC, are involved in the cognitive modulation of pain. Previous multimodal MRI studies have demonstrated reductions in cortical thickness and spontaneous brain activity in these dopaminergic regions among PD patients with persistent pain compared to nPDP patients (Polli et al., 2016). The cerebellum, via its connections with midbrain dopaminergic regions as well as sensorimotor, emotional, and cognitive neworks, plays a modulatory role in pain processing (Flace et al., 2021; Li et al., 2024). Studies have observed abnormal cerebellar activation in PD patients with persistent pain (Polli et al., 2016) and in pain-free PD patients during thermal stimulation (Tessitore et al., 2017). Hence, our findings of reduced FC between the putamen and these pain-related regions in PDP patients may indicate impaired dopaminergic transmission within the nigrostriatal-cortical pathway, contributing to abnormal pain perception and modulation in PD.

This study has several limitations that should be acknowledged. First, the cross-sectional design of the study limits the ability to establish causality between the observed brain alterations and pain in PD. Future longitudinal studies are necessary to clarify this causal relationship. Second, to minimize the effects of medication on brain function, all participants were instructed to stop their medication 12 h prior to the scan. However, this could potentially increase their pain levels and alter brain function. Third, relying solely on VAS scores to assess pain intensity limited the exploration of other pain dimensions and their underlying neural mechanisms in PD.

## Conclusion

In conclusion, this study demonstrates that chronic pain in PD is associated with reduced ALFF in the putamen and disrupted FC with brain regions involved in pain perception and modulation, highlighting the critical role of dopaminergic degeneration in the development and maintenance of pain in PD. Furthermore, the increased ALFF in the motor regions of PDP patients may reflect ineffective compensatory mechanisms attempting to suppress pain. Our findings may provide theoretical evidence supporting the efficacy of dopaminergic medications in alleviating PD-related pain and identify novel therapeutic targets for neuromodulation therapy, such as the putamen and M1 cortex.

## Data Availability

The raw data supporting the conclusions of this article will be made available by the authors, without undue reservation.

## Footnotes

^1^ http://www.rfmri.org/dpabi
